# Birds of primary and secondary forest and shrub habitats in the peat swamp of Berbak National Park, Sumatra

**DOI:** 10.12688/f1000research.13996.2

**Published:** 2018-05-14

**Authors:** Kevin Darras, Dedi Rahman, Waluyo Sugito, Yeni Mulyani, Dewi Prawiradilaga, Agus Rozali, Irfan Fitriawan, Teja Tscharntke

**Affiliations:** 1 Department of Crop Sciences, Agroecology, University of Goettingen, Göttingen, 37077, Germany; 2 Zoological Society of London, Jambi, Sumatra, Indonesia; 3 Department of Forest Resources Conservation and Ecotourism, Faculty of Forestry, Bogor Agricultural University, Bogor, Indonesia; 4 Research Centre for Biology LIPI, Cibinong Science Centre, Bogor, Indonesia; 5 Research Staff of CRC 990, Faculty of Agriculture, University of Jambi, Jambi, Indonesia; 6 Conservation Staff, PT Henrison Inti Persada, Sorong, West Papua , Indonesia

**Keywords:** primary forest, secondary forest, shrub swamp, swamp forest, community ecology, forest disturbance, forest fires, selective logging

## Abstract

**Background:** Tropical lowland rainforests are threatened by deforestation and degradation worldwide. Relatively little research has investigated the degradation of the forests of South-east Asia and its impact on biodiversity, and even less research has focused on the important peat swamp forests of Indonesia, which experienced major losses through severe fires in 2015.

**Methods:** We acoustically sampled the avifauna of the Berbak National Park in 2013 in 12 plots split in three habitats: primary swamp forest, secondary swamp forest, and shrub swamp, respectively representing non-degraded, previously selectively logged, and burned habitats. We analysed the species richness, abundance, vocalisation activity, and community composition across acoustic counts, plots, feeding guilds and IUCN Red List categories. We also analysed community-weighted means of body mass, wing length, and distribution area.

**Results:** The avifauna in the three habitats was remarkably similar in richness, abundance and vocalisation activity, and communities mainly differed due to a lower prevalence of understory insectivores (Old-World Babblers, Timaliidae) in shrub swamp. However primary forest retained twice as many conservation-worthy species as shrub swamp, which harboured heavier, probably more mobile species, with larger distributions than those of forest habitats.

**Conclusions:** The National Park overall harboured higher bird abundances than nearby lowland rainforests. Protecting the remaining peat swamp forest in this little-known National Park should be a high conservation priority in the light of the current threats coming from wildlife trade, illegal logging, land use conversion, and man-made fires.

## Introduction

We are losing tropical forests and their associated biodiversity worldwide to deforestation, and this loss is irreplaceable ^1^. Forest loss also occurs because of degradation: selective logging affects large areas ^2^, which in turn become more susceptible to other disturbances like fire ^3^. Most studies focus on the Amazon region, but forests in Southeast Asia also face great threats ^4^. Forest losses due to fire have recently gained more attention in Indonesia ^5, 6^. Indonesia has the highest deforestation rate of all countries ^7^, and even protected forests suffer losses ^8^. The effectiveness of protecting forests in Indonesia has been questioned for Kalimantan ^9^, but for Sumatra, modest progress has been made, especially as large-scale logging has slowed down ^10^.

The impacts of forest loss and degradation on biodiversity are better known in the Amazon region, for instance for birds ^11^. In Southeast Asia, most primary forest bird species still occur in previously logged forests ^12– 14^ (Azhar *et al*. 2011). Globally, bird feeding guilds respond differently to disturbance ^15^, but forest understory insectivores were identified as the most sensitive ^16^. The severity of the disturbance and its impact on soils largely determine the duration of recovery, while the surrounding landscape acts as a source of biota ^17^

We lack studies investigating the impact of disturbances - from logging or fire - on bird communities in peat swamp forests, despite their crucial importance for biodiversity, flood control, carbon stocks, potential greenhouse gas emissions, and their high vulnerability to drainage and fires ^18, 19^. Theoretical and modelling approaches are usually used to analyse the potential benefit of disturbances for overall landscape biodiversity ^20, 21^.

In this paper we describe the bird communities in a tropical peat swamp on Sumatra, Indonesia. Bird surveys of the Berbak area date far back ^22, 23^. We sampled birds in three habitats defined according to Keddy (2010) ^24^: primary swamp forest, secondary swamp forest resulting from selective logging, and shrub swamp resulting from forest fire. We compare species richness and bird communities between these habitats both taxonomically (species richness, abundance) and functionally (vocalisation activity, body mass, wing length, distribution area). We ask the question whether disturbances increase the overall diversity of the landscape. We discuss the implications of our results for maintaining the overall bird diversity of the peat swamp and for bird conservation in Berbak.

## Methods

### Study site

**Figure 1.**
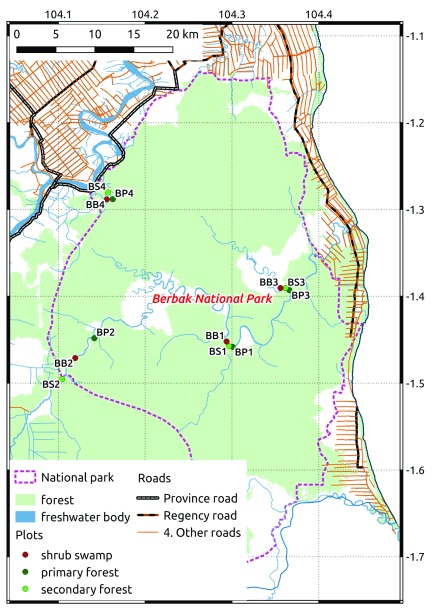
Map of the study area.

We surveyed birds in Berbak National Park, a peat swamp situated in the province of Jambi, on the east coast of the island of Sumatra in Indonesia ( Figure 1). Berbak National Park is a Ramsar site ^25^ and an Important Bird Area ^26 ^. A severe drought in 1997 facilitated forest fires which were aided by human disturbance due to natural rubber collection ( *Dyera costulata*). The burned areas subsequently developed into shrub swamp. Tree stumps also reveal that illegal selective logging affected several areas of the park, resulting in secondary forest habitats.

We chose 12 plots for counting birds, divided in four sites for each of three habitat types of the peat swamp area, representing different past disturbance levels: primary forest (no disturbance), secondary forest (selective logging), and shrub swamp (fire). The secondary forest plots were last subject to selective logging until 2008 for BS1, 1999 for BS2, 2003 for BS3, and 2010 for BS4. The shrub swamp plots burned in 1997, and BB2 burned yearly since.

***Field surveys.*** We recorded audible sound in all 12 plots two times. We used autonomous sound recorders (SM2+ recorders with SMX-II microphones, *Wildlife Acoustics Inc.*) and did not carry out visual surveys, since acoustic recording constitutes a valid survey method for assessing bird richness ^27^, especially for cryptic birds in tropical forests. From February to November 2013, we sampled all three plots of one site each month for 48 hours, starting at midnight. It was not possible to conduct the survey each month, or to repeat the same plot sampling sequence for the second set of recordings because of access and transportation restrictions. Before the recording started, we counted and measured the diameter at breast height (DBH) of all trees with a circumference above 20 cm (diameter at breast height of ~6.4 cm) inside an area of 14 × 14 m delimited by spanning 10 m coloured ropes from the central tree where the sound recorder was attached to all cardinal directions. We also recorded whether the plot was flooded or not at the time of the sound recorder installation.

### Data analysis

We uploaded 20 minutes recordings starting at sunrise from each plot from each month (24 recordings in total, 2 per plot) to our online platform ( http://soundefforts.uni-goettingen.de/). The provenance of the recordings was hidden and all birds within the sound recordings were identified by author IF. The distance of bird vocalisations was estimated by ear to the meter; even though the accuracy is lower, estimation error was assumed to be random. Distance estimates were made based on the call loudness in the sound recording compared to the ambient sound level and knowledge of the source sound level of each species. The start and end time of each vocalization was recorded to compute the duration of the bird vocalisations. We complemented our data set with species-specific information about the feeding guild ^28, 29^, body mass data ^28, 30^, wing length data ^31^, the IUCN Red List threat status ^32^ and distribution area ^33^ to analyse conservation and functional aspects of bird community differences.

The data were analysed in R 3.4.3 and graphs were generated with the package ggplot2 ^34^. We excluded detections that were not identified to species, as well as detections above 50 m to compare plots at a common detection radius ^35^. We computed species richness, bird vocalizing activity in minutes, and bird abundance per acoustic count (i.e. recording). Bird species richness was further computed at the plot (alpha richness) and habitat (gamma richness) levels. For counting bird abundance, we first derived the maximum number of simultaneously vocalising individuals in each species, and then summed these maxima over all species, leading to a conservative estimate of the number of individuals per count. We calculated community-weighted means (hereafter CWM, also called community functional parameter ^36^) for body mass, wing length, and distribution area for each count. Due to microphone failure, the second recording of one of the forest plots had only one audible audio channel and was therefore removed from the count-level analysis.

At the count level, the species richness and abundance between habitats was modelled using generalised linear mixed effects models of the poisson family (lme4 package ^37^), with plot as random variable.. Similarly, vocalizing activity was analysed with a linear mixed effects model. We checked that the models were not over-dispersed and that the standardised residuals did not indicate heteroscedasticity. At the plot level, we used generalised linear models of the poisson family to model alpha and beta bird species richness, which we calculated using the additive partitioning approach ^38^: alpha richness was the mean number of species per plot, and beta richness was defined as the total number of species in the plot’s habitat (gamma richness) minus its alpha diversity. For all models, we used tree number, tree basal area, and habitat type as predictors. We generated all possible predictor combinations and compared the models using Akaike's Information Criterion for small sample sizes (herafter AICc, MuMIn package, dredge function) to choose the best model (with the lowest AICc).

We pooled the data from both acoustic counts and visualized the composition of the bird communities in non-metric multidimensional scaling graphs generated with the package vegan ^39^, and tested the significance of the habitat in structuring these communities with an ADONIS test ^40^. We also plotted the abundance of birds within different families in each of the habitats along with their conservation status. To investigate whether the combined, different habitats lead to higher species richness than one primary forest area of similar size, we calculated the rarefied richness based on the entire bird community, rarefied to 4 sampling plots, to compare it to the number of species found in the 4 forest plots.

## Results

We detected 426 birds overall. Among those, 30 individuals were not identified to species level and 2 were detected above 50 m, resulting in a working dataset of 394 individuals, belonging to 88 species ( Table S1). The three habitats differed considerably based on their vegetation structure ( Figure S1) and the distribution of their DBH values, with primary forest having the highest basal area, secondary forest intermediate values, and shrub swamp having the smallest basal area ( Figure S2).

**Figure 2.**
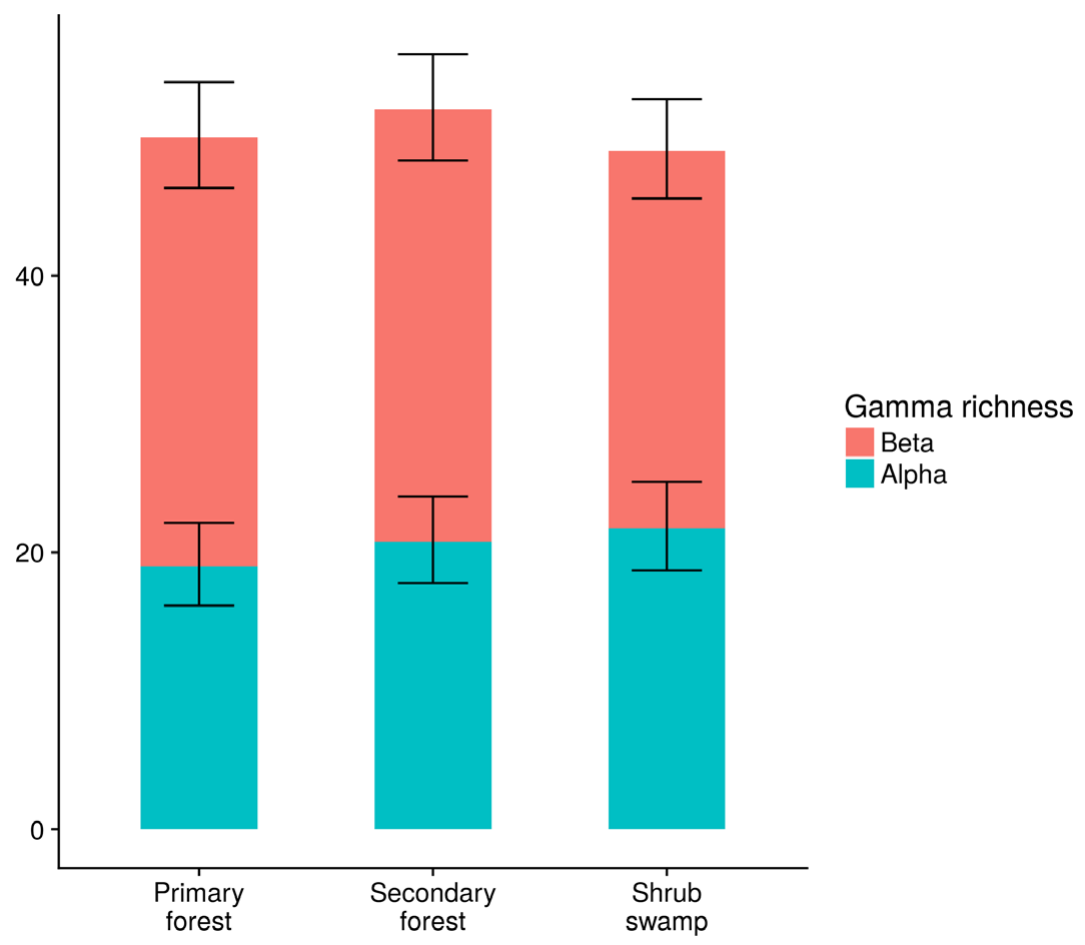
Bird richness components at the plot scale.

**Figure 3.**
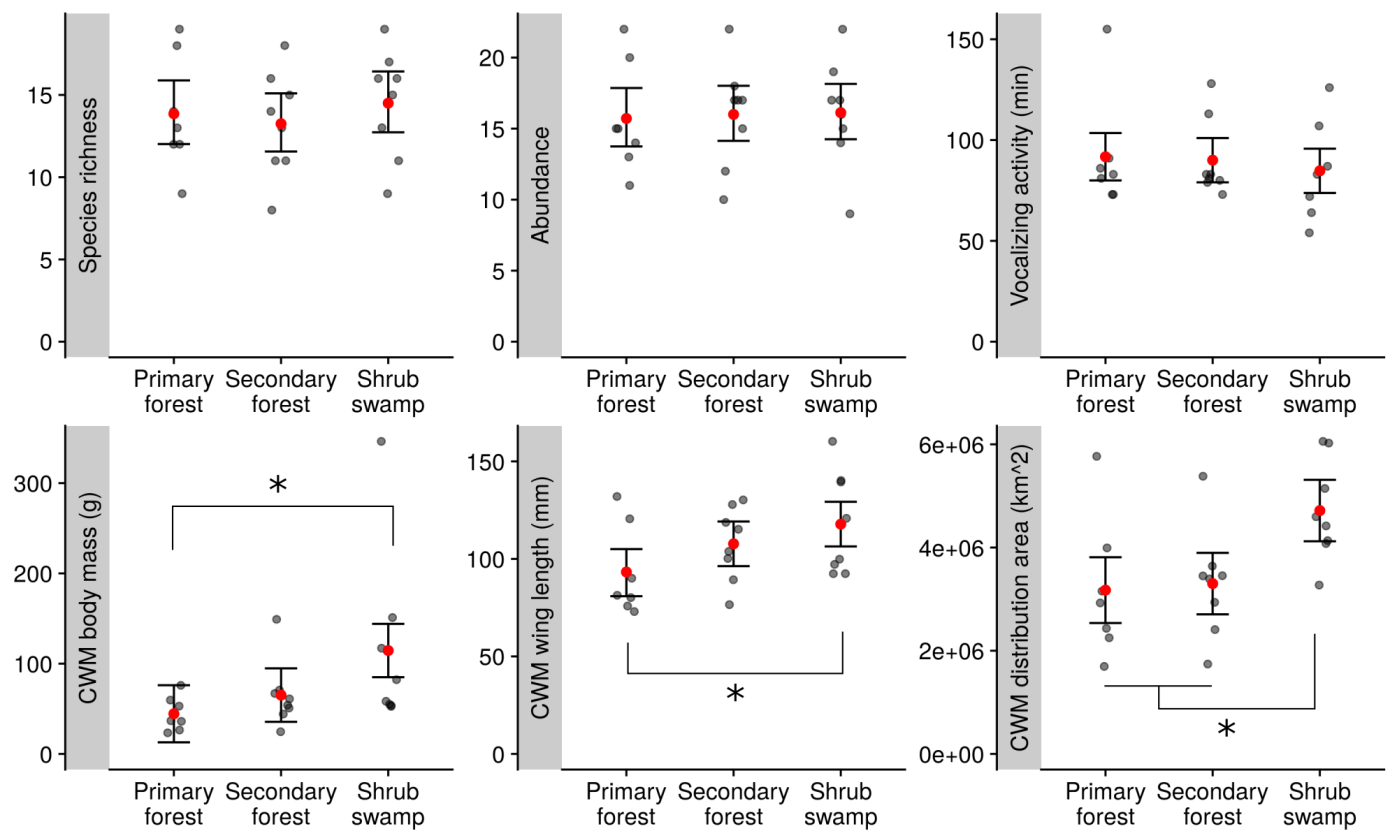
Bird taxonomical and functional traits in each habitat.

Species richness and abundance at the count level, and mean alpha and beta species richness at the plot level were similar between the habitats ( Figure 2 and Figure 3).

Total (gamma) richness for each habitat type, split by alpha and beta richness components. Error bars show the 83% confidence intervals for the mean alpha and beta richness values, which do not differ significantly at P=0.05 when they overlap (Krzywinski, 2013). 

These variables were best explained by null models. Gamma species richness per habitat was as follows: primary forest: 50 species, secondary forest: 52 species, bush swamp: 50 species. Bird richness and abundance between habitats, split into different functional groups and IUCN red list threat categories, were similar between habitats ( Figure S2).

Bird vocalisation activity at the count level was similar among habitats ( Figure 3) and best explained by a null model. CWMs of body mass, wing length and distribution area however were increasing along the disturbance gradient and the CWMs in shrub swamp were significantly higher than in primary forest. Wing length CWM was also higher in shrub swamp than in secondary forest ( Figure 3).

Bird species richness, abundance, vocalizing activity, community-weighted mean (CWM) of body mass, wing length, and distribution area ^33^ per count in three different habitats of the peat swamp of the Berbak national park. Mean values are represented with red dots and their 83% confidence intervals are indicated with error bars. Means are significantly different at P=0.05 when their confidence intervals do not overlap (Krzywinski, 2013), and significant differences are indicated with asterisks.

**Figure 4.**
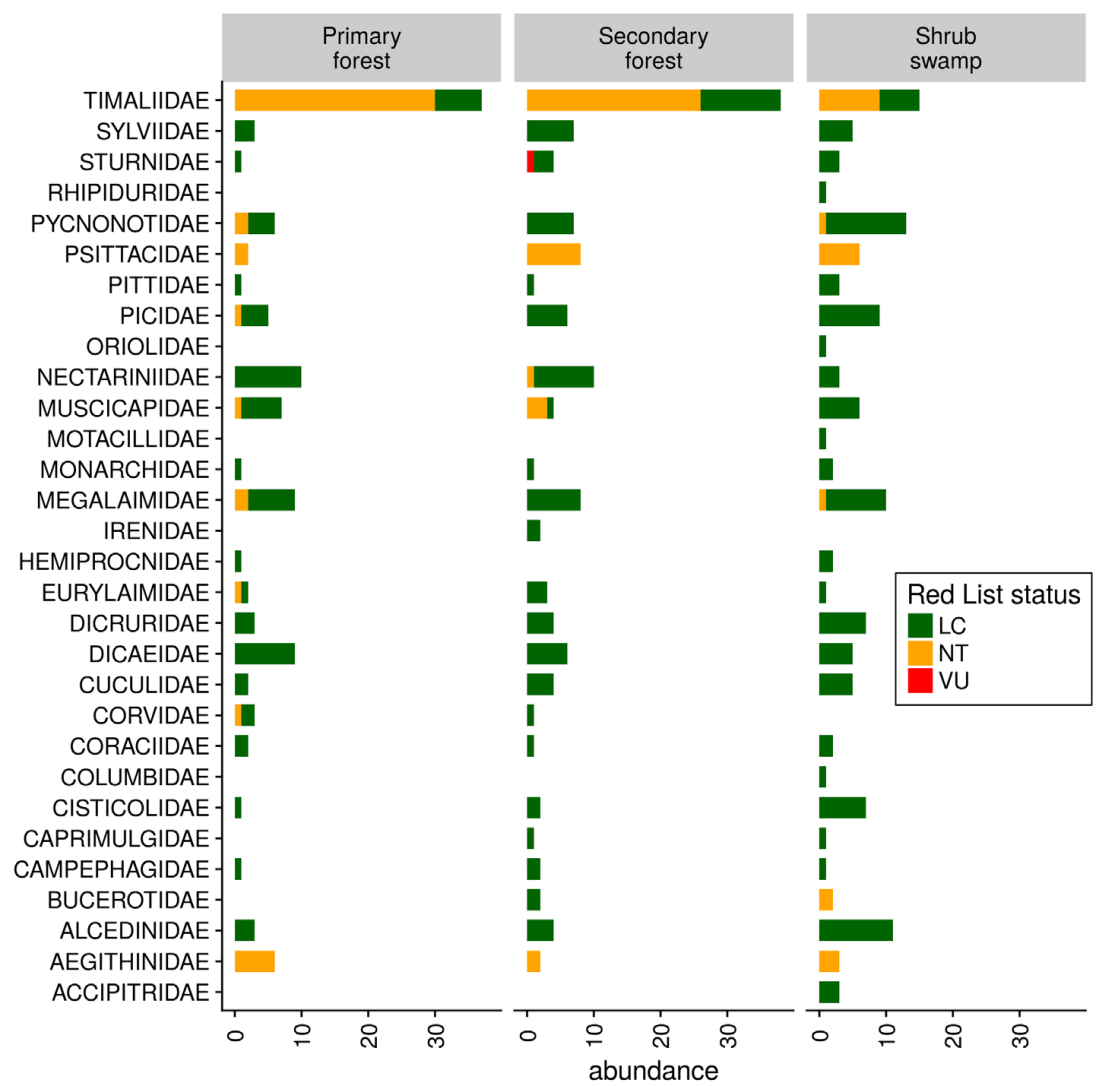
Bird abundance in each family.

Bird communities differed greatly between primary forest and bush swamp, with secondary forest being an intermediate habitat having overlap with both of the latter ( Figure S3). The ADONIS test revealed that habitat structured bird community composition with marginal significance (P=0.068). The difference in the bird communities arose mainly from the higher abundance of Timaliidae (Old World babblers) in the forest habitats. Pycnonotidae (Bulbuls) and Alcedinidae (Kingfishers) were more prevalent in shrub swamp, while Nectarinidae were more frequent in the forest habitats ( Figure 4).

Abundances are split by habitat and shown for different IUCN Red List statuses.

The data and R script required to reproduce our graphs and results are provided This file contains: Berbak birds.csv contains the bird detection data; Berbak vegetation.csv contains the individual DBH values for all plots; Berbak birds analysis.R is the R script that performs the analysis and graphing. Click here for additional data file. Copyright: © 2018 Darras K et al. 2018 Data associated with the article are available under the terms of the Creative Commons Zero "No rights reserved" data waiver (CC0 1.0 Public domain dedication).

## Discussion

We have shown that all three habitats inside the Berbak peat swamp forest have similar abundance, vocalizing activity and species richness. The habitat, tree number or basal area were not related to these measures. Even the richness and abundance of the bird feeding guilds and abundances inside families were similar between habitats. However, we detected differences in the bird community composition, notably a decrease in understory insectivores (Old World Babblers, Timaliidae) which was compensated by the appearance of other species in shrub swamp. This change in the community also became apparent in a shift to bigger and more mobile species with greater distribution ranges in shrub swamp.

The Berbak peat swamp forest is threatened from many sides. It is difficult to access as only waterways (irrigation canals and rivers) lead into it, and large parts are temporarily flooded due to tides. However, wild bird extraction for the caged bird market, illegal logging, land-use conversion at its margins, and natural rubber (jelutung) collection are all currently happening ^41^. Wild bird trapping threatens bird populations directly (Harris *et al*. 2016), and is especially worrisome as birds from the Berbak region are increasingly traded in the caged bird market of Jambi city (pers. obs. KD). All these human activities increase the risk of fires, especially during dry spells caused by the warm phases of the El Niño Southern Oscillation, which led to the especially severe fires of 1994, 1997, and 2015 (after our survey).

Primary forest had high conservation value because we found 16 species of conservation concern ("near threatened" status according to IUCN Red List status), twice as many as in shrub swamp. More acutely threatened, rare species might be detected with higher sampling effort by processing more recordings. Secondary forest was exactly intermediary with 12 species of conservation concern, although one bird, the Javan Myna ( *Acridotheres javanicus*), has recently been classified as vulnerable. Javan Mynas are commonly sold on the market in Jambi city and might establish feral populations when occasionally breaking free. The absence of hornbills in primary forest seems fortuitous, as we also detected one *Buceros rhinoceros* in forest also, but at an estimated distance of 60 m. Compared to the secondary forest plots surveyed by Prabowo *et al*. ^42^ in the same province (Harapan rainforest) and year, the detected bird abundance was much higher in Berbak (4 birds per count in Prabowo *et al*. versus 13 birds per count in the present study for the same detection radius). Our secondary forest plots seemed relatively well-preserved, as we could not detect any typical loss of understory and terrestrial insectivores due to changes in understory vegetation ^43^. It is noteworthy that even extensively logged peat swamp forest can harbor large species numbers. (Azhar *et al*. 2011). Considering that the detected abundance of birds in the other habitats was similarly high, this indicates that the National Park of Berbak still provides relatively good living conditions for birds, and especially conservation-worthy species thrive in the primary forest tracts.

Nevertheless, bird communities differ between habitats as was shown in the non-metric multidimensional analysis ( Figure S4). It turned out that the differences in bird communities arose mainly from the absence of Timaliidae in shrub swamp. Timaliidae are mainly understory insectivores, which are generally recognised to be sensitive to forest disturbance and thus act as indicator species ^44^. The higher prevalence of generalist bulbuls and open-area kingfishers in shrub swamp is also typical of disturbed habitats in the region, such as oil palm plantations ^42^. Notably, sunbirds were almost absent in shrub swamp, which does not seem to provide enough floral resources (pers. obs. KD). Interestingly, the species in shrub swamp were heavier, had longer wings and wider distribution ranges, indicating they may be more mobile, widespread species that are less of a conservation concern; these features are also typical of generalist species. The higher body masses may arise from the prevalence of kingfishers and bulbuls, which are relatively big.

## Conclusion and outlook

The different habitats lead to a high diversity at the national park level: while the total species count per habitat was almost identical (around 50), the overall species richness reached 88 species. It is tempting to conclude that the heterogeneity introduced by disturbances such as logging or fires (which created these different habitats) increased bird species richness. However, in comparison to an equally large area consisting only of primary forest, the combination of different habitats does not lead to a markedly higher species richness, as we found a nearly identical rarefied species richness of 51 for all habitats combined. The fact also remains that primary forest harboured a higher proportion of conservation-worthy bird species, while generalists were more prevalent in the shrub swamp.

Our autonomous acoustic sampling protocol gathered many more data than we analysed so far: we only processed around 0.7% of the available data, albeit the most promising dawn choruses. Also, due to logistical restrictions, we only surveyed four sites that were close to waterways, so that that large parts of the park remain little known. We would determine a higher proportion of the bird community with random or carefully chosen time windows throughout the day ^45^. We welcome interested prospective co-authors to process more recordings to complete the species list of the still only superficially studied avifauna of Berbak. Repeated surveys to the same sites, after the major fires from 2015, would also yield insights into how bird populations are changing in the longer term in response to these and other disturbances from wildlife trade.

## Data availability

The data referenced by this article are under copyright with the following copyright statement: Copyright: © 2018 Darras K et al.

Data associated with the article are available under the terms of the Creative Commons Zero "No rights reserved" data waiver (CC0 1.0 Public domain dedication). 

**Dataset 1: The data and R script required to reproduce our graphs and results are provided**. This file contains: Berbak birds.csv contains the bird detection data; Berbak vegetation.csv contains the individual DBH values for all plots; Berbak birds analysis.R is the R script that performs the analysis and graphing. DOI, 10.5256/f1000research.13996.d203364 ^46^.

The source audio material is available at http://soundefforts.uni-goettingen.de/biosounds/collection/show/3.

## Supplementary material

**Table S1: Detected bird numbers in each habitat.**

**Figure S1: Examples of sampling plots.**

1: BB3 shrub swamp with burned tree stump; 2: BP2 primary swamp forest; 3: BS2: secondary swamp forest with installed sound recorder and coloured ropes for the vegetation survey.

**Figure S2: Distribution of tree sizes in the different habitats.**

We measured the diameter at breast height (DBH) of all trees with a DBH above approximately 6.4 cm (circumference of 20 cm).

**Figure S3: Species richness per habitat and IUCN Red List threat status.**

**Figure S4: Non-metric multidimensional scaling of bird communities in the different habitats.**

Brown represents shrub swamp, light green secondary forest, dark green primary forest.
