# Supplementary material for: Birds of primary and secondary forest and shrub habitats in the peat swamp of Berbak National Park, Sumatra
Source: F1000Res. 2018 May 14;7:229. Originally published 2018 Feb 26. [Version 2] doi: 10.12688/f1000research.13996.2 (PMC6058469; doi:10.12688/f1000research.13996.2)
Supplement: Supplementary file 2 [file f1000research-7-16242-s0001.tgz › d59ff5b2-dce8-47dd-bc30-17bd51983639.docx]

**Table S1: Detected bird numbers in each habitat.**

| **Species** | **Primary forest** | **Secondary forest** | **Shrub swamp** |
| --- | --- | --- | --- |
| *Acridotheres javanicus* | 0 | 1 | 0 |
| *Aegithina viridissima* | 6 | 2 | 3 |
| *Aethopyga siparaja* | 1 | 2 | 1 |
| *Aethopyga temminckii* | 1 | 0 | 0 |
| *Anthracoceros albirostris* | 0 | 2 | 0 |
| *Anthreptes malacensis* | 1 | 1 | 0 |
| *Anthreptes rhodolaemus* | 0 | 1 | 0 |
| *Arachnothera hypogrammica* | 4 | 2 | 2 |
| *Arachnothera longirostra* | 3 | 4 | 0 |
| *Brachypodius atriceps* | 0 | 0 | 1 |
| *Buceros rhinoceros* | 0 | 0 | 2 |
| *Cacomantis merulinus* | 0 | 0 | 1 |
| *Caprimulgus macrurus* | 0 | 1 | 1 |
| *Centropus bengalensis* | 0 | 0 | 2 |
| *Centropus sinensis* | 1 | 1 | 1 |
| *Chrysococcyx xanthorhynchus* | 1 | 0 | 0 |
| *Chrysophlegma miniaceum* | 0 | 2 | 3 |
| *Copsychus saularis* | 0 | 0 | 1 |
| *Corvus enca* | 2 | 0 | 0 |
| *Corydon sumatranus* | 0 | 1 | 0 |
| *Cyanoderma erythropterum* | 5 | 9 | 1 |
| *Cyanoderma rufifrons* | 0 | 1 | 0 |
| *Cymbirhynchus macrorhynchos* | 0 | 1 | 1 |
| *Cyornis concretus* | 1 | 0 | 0 |
| *Cyornis olivaceus* | 0 | 1 | 0 |
| *Cyornis rufigastra* | 4 | 0 | 4 |
| *Cyornis turcosus* | 1 | 2 | 0 |
| *Dendronanthus indicus* | 0 | 0 | 1 |
| *Dicaeum cruentatum* | 1 | 0 | 0 |
| *Dicaeum trigonostigma* | 8 | 6 | 5 |
| *Dicrurus aeneus* | 0 | 1 | 0 |
| *Dicrurus paradiseus* | 3 | 3 | 6 |
| *Dryocopus javensis* | 0 | 0 | 1 |
| *Euptilotus eutilotus* | 1 | 0 | 0 |
| *Eurylaimus harterti* | 0 | 1 | 0 |
| *Eurylaimus ochromalus* | 1 | 0 | 0 |
| *Eurystomus orientalis* | 2 | 1 | 2 |
| *Ficedula zanthopygia* | 0 | 0 | 1 |
| *Gracula religiosa* | 1 | 3 | 3 |
| *Halcyon smyrnensis* | 0 | 1 | 5 |
| *Hemiprocne longipennis* | 1 | 0 | 2 |
| *Hemipus hirundinaceus* | 1 | 0 | 1 |
| *Hierococcyx fugax* | 0 | 0 | 1 |
| *Hydrornis guajanus* | 0 | 1 | 1 |
| *Hypothymis azurea* | 1 | 1 | 2 |
| *Iole charlottae* | 1 | 0 | 0 |
| *Irena puella* | 0 | 2 | 0 |
| *Lacedo pulchella* | 1 | 0 | 0 |
| *Leptocoma sperata* | 0 | 0 | 1 |
| *Macronus ptilosus* | 4 | 5 | 5 |
| *Malacopteron affine* | 9 | 8 | 0 |
| *Malacopteron magnum* | 1 | 1 | 0 |
| *Meiglyptes tukki* | 1 | 0 | 0 |
| *Micropternus brachyurus* | 3 | 3 | 5 |
| *Mixornis gularis* | 1 | 2 | 5 |
| *Oriolus chinensis* | 0 | 0 | 1 |
| *Orthotomus atrogularis* | 3 | 4 | 1 |
| *Orthotomus ruficeps* | 0 | 1 | 1 |
| *Orthotomus sericeus* | 0 | 2 | 3 |
| *Pelargopsis capensis* | 2 | 3 | 6 |
| *Pericrocotus flammeus* | 0 | 2 | 0 |
| *Philentoma pyrhoptera* | 1 | 0 | 0 |
| *Picus puniceus* | 1 | 0 | 0 |
| *Pitta sordida* | 1 | 0 | 2 |
| *Platylophus galericulatus* | 1 | 0 | 0 |
| *Platysmurus leucopterus* | 0 | 1 | 0 |
| *Prinia familiaris* | 1 | 1 | 0 |
| *Prinia flaviventris* | 0 | 1 | 7 |
| *Psilopogon chrysopogon* | 0 | 0 | 1 |
| *Psilopogon duvaucelii* | 6 | 6 | 8 |
| *Psilopogon mystacophanos* | 2 | 0 | 1 |
| *Psittacula longicauda* | 1 | 5 | 6 |
| *Psittinus cyanurus* | 1 | 3 | 0 |
| *Pycnonotus cyaniventris* | 0 | 0 | 1 |
| *Pycnonotus goiavier* | 1 | 1 | 3 |
| *Pycnonotus plumosus* | 1 | 5 | 8 |
| *Pycnonotus simplex* | 1 | 1 | 0 |
| *Rhinortha chlorophaea* | 0 | 2 | 0 |
| *Rhipidura javanica* | 0 | 0 | 1 |
| *Sasia abnormis* | 0 | 1 | 0 |
| *Serilophus lunatus* | 1 | 0 | 0 |
| *Spilornis cheela* | 0 | 0 | 3 |
| *Stachyris maculata* | 6 | 6 | 3 |
| *Stachyris nigricollis* | 3 | 2 | 1 |
| *Stachyris poliocephala* | 1 | 0 | 0 |
| *Surniculus lugubris* | 0 | 1 | 0 |
| *Treron vernans* | 0 | 0 | 1 |
| *Trichastoma malaccense* | 7 | 0 | 0 |
| *Trichixos pyrropygus* | 0 | 1 | 0 |
