# Supplementary figures and images for: Birds of primary and secondary forest and shrub habitats in the peat swamp of Berbak National Park, Sumatra
Source: F1000Res. 2018 May 14;7:229. Originally published 2018 Feb 26. [Version 2] doi: 10.12688/f1000research.13996.2 (PMC6058469; doi:10.12688/f1000research.13996.2)

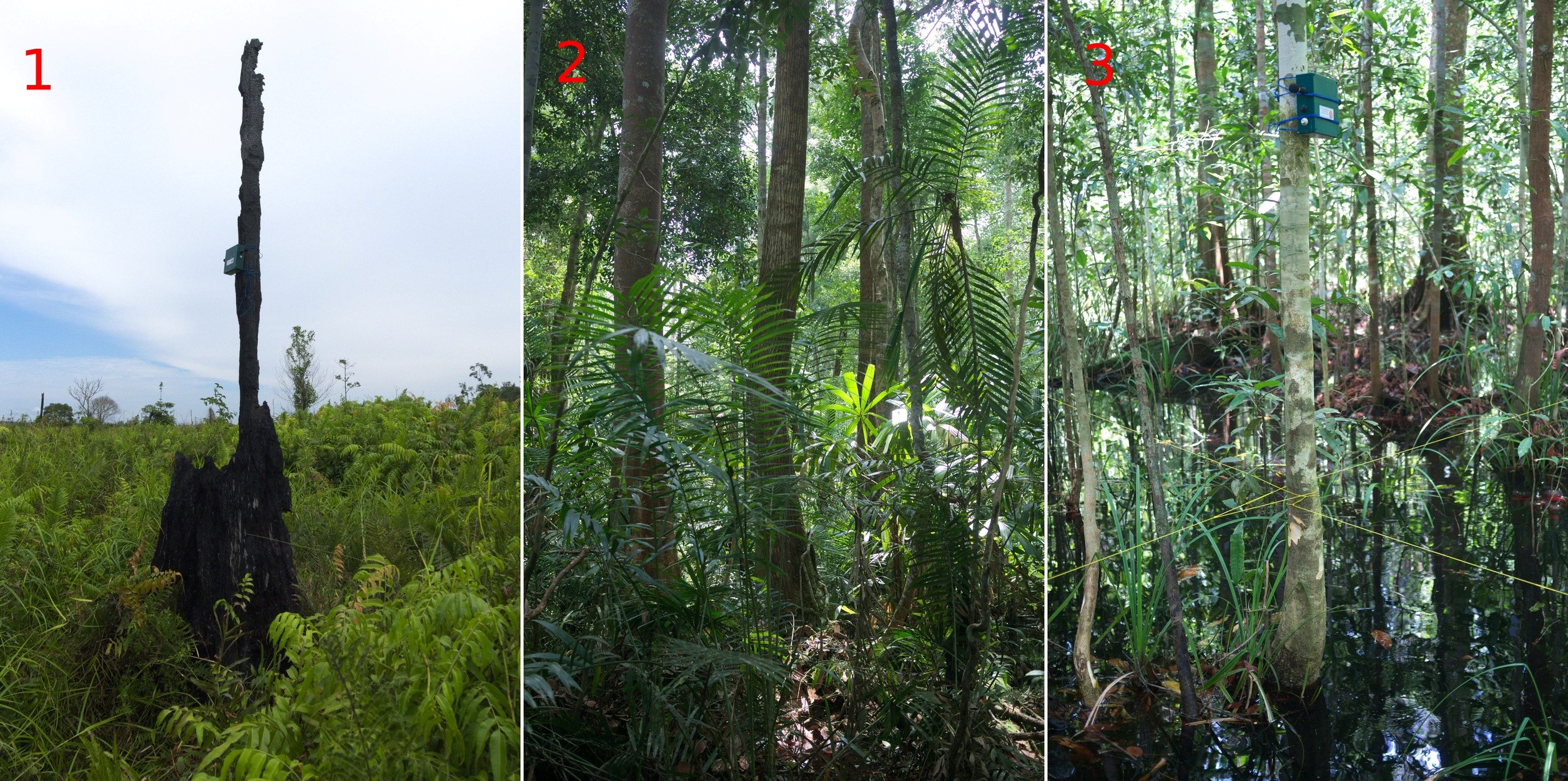

Supplement: Supplementary file 3 [file f1000research-7-16242-s0002.tgz › 86129e6e-e7b0-4522-9e62-04324621c94b.jpg]

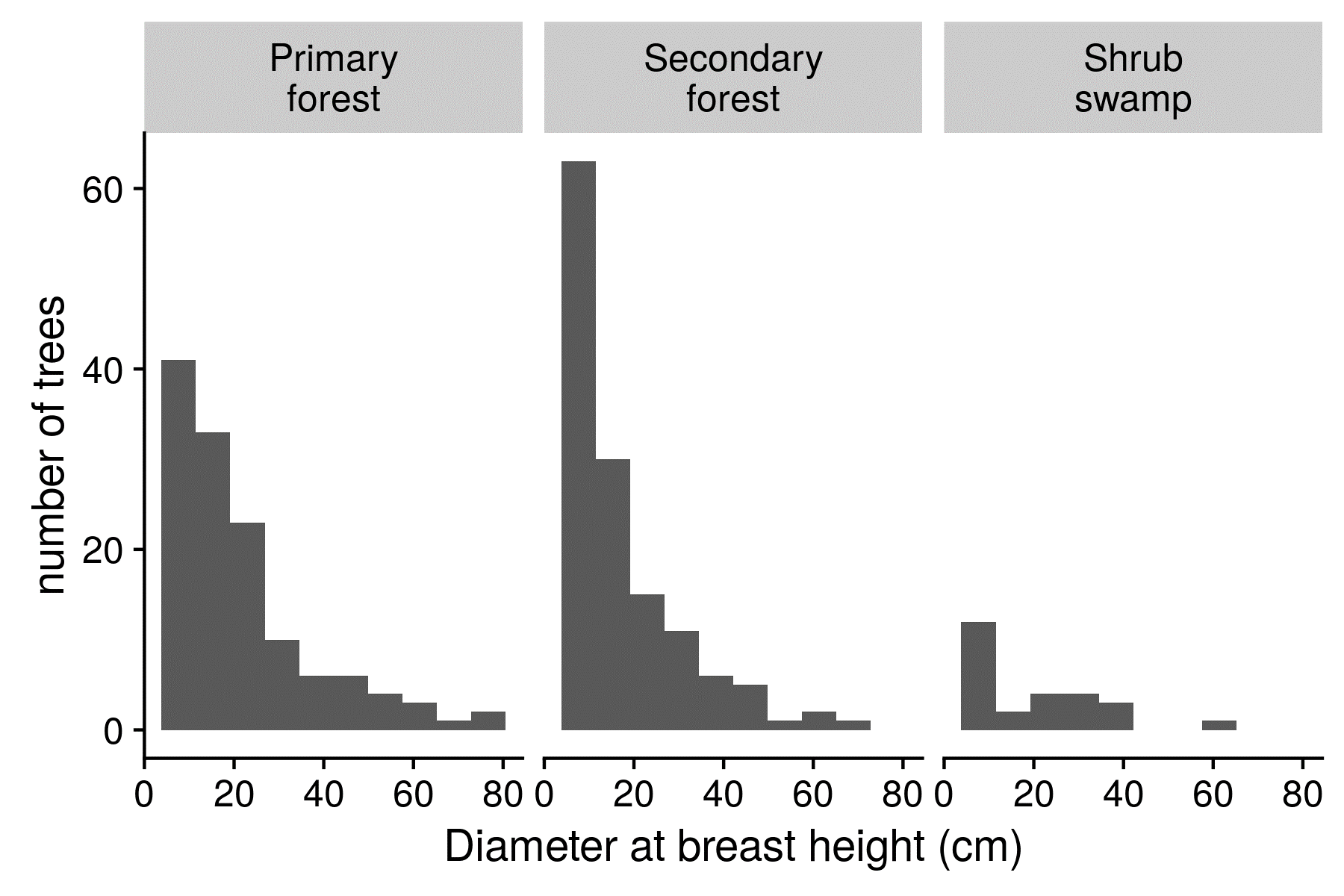

Supplement: Supplementary file 4 [file f1000research-7-16242-s0003.tgz › 046cdfaf-c8ae-45ab-aeab-c5c6f6cdea9d.png]

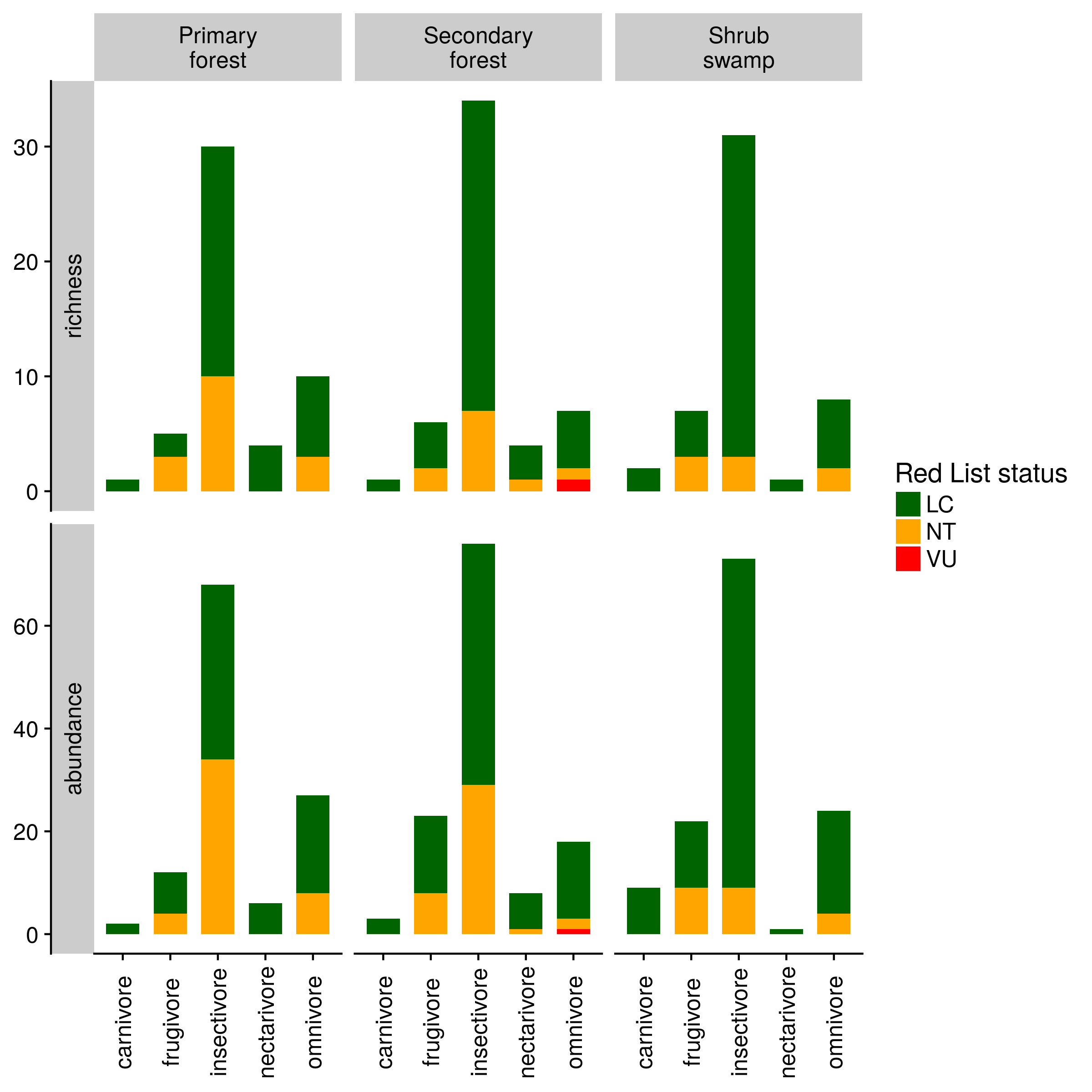

Supplement: Supplementary file 5 [file f1000research-7-16242-s0004.tgz › 6bbc3967-ea93-4abf-8f2a-cb256f675aba.png]

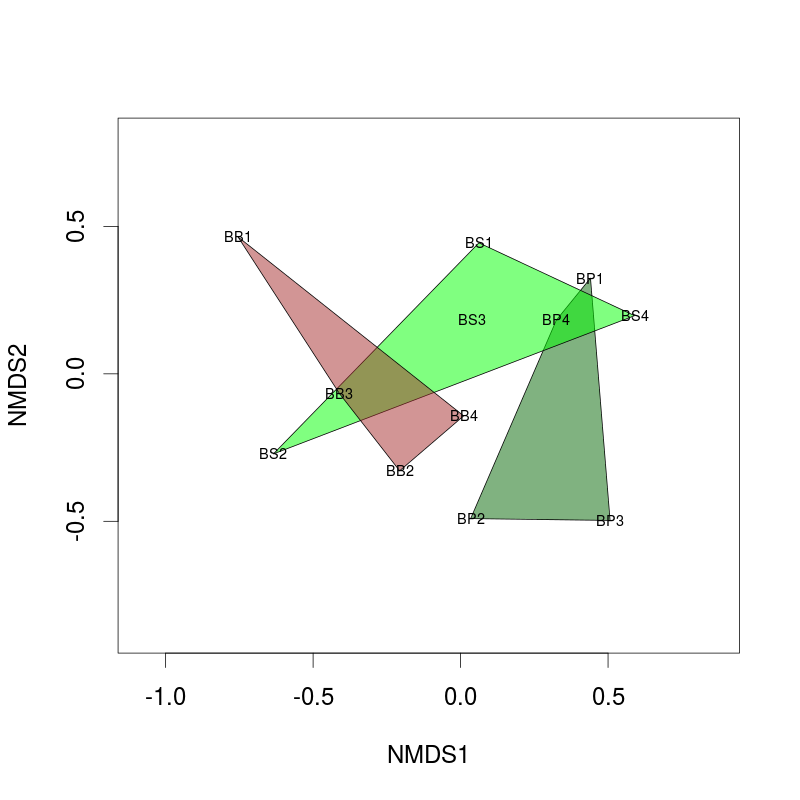

Supplement: Supplementary file 6 [file f1000research-7-16242-s0005.tgz › 47c7410d-67be-4c90-9d73-5c83e601a310.png]
