# Supplementary material for: Birds of primary and secondary forest and shrub habitats in the peat swamp of Berbak National Park, Sumatra
Source: F1000Res. 2018 May 14;7:229. Originally published 2018 Feb 26. [Version 2] doi: 10.12688/f1000research.13996.2 (PMC6058469; doi:10.12688/f1000research.13996.2)
Supplement: Peer review history

# Peer review: Referee response for version 2

**DOI:** 10.5256/f1000research.16242.r33970

**Published:** 2018-07-23

**Authors:** Erin M. Bayne

**Affiliations:** 1 Department of Biological Sciences, University of Alberta, Edmonton, AB, Canada

The authors have addressed my primary concerns and the manuscript is much clearer now.

I have read this submission. I believe that I have an appropriate level of expertise to confirm that it is of an acceptable scientific standard.

# Peer review: Referee response for version 2

**DOI:** 10.5256/f1000research.16242.r33969

**Published:** 2018-05-21

**Authors:** Badrul Azhar

**Affiliations:** 1 Faculty of Forestry, Institute of Bioscience, Universiti Putra Malaysia, Serdang, Malaysia

The authors have addressed all the major comments. I'm satisfied with the explanations and justifications given by the authors.

I have read this submission. I believe that I have an appropriate level of expertise to confirm that it is of an acceptable scientific standard.

# Response

**Published:** 2018-05-21

**Authors:** Kevin Darras

**Affiliations:** University of Goettingen, Germany

Thank you for your time and interest!

# Peer review: Referee response for version 1

**DOI:** 10.5256/f1000research.15212.r32057

**Published:** 2018-04-04

**Authors:** Erin M. Bayne

**Affiliations:** 1 Department of Biological Sciences, University of Alberta, Edmonton, AB, Canada

The article "Birds of primary and secondary forest and shrub habitats in the peat swamp of Berbak National Park, Sumatra" by Darras et al. summarizes a survey of birds via autonomous recording units in this poorly understood ecosystem. Any information on systems such as these is valuable and providing the type of data given is useful for providing baseline information that give us an idea of what species are present and thus at risk. Thus, the data as shared is very valuable for the scientific and conservation community.

 The introduction is fairly generic in my opinion and focuses on issues that are not the central tenet of the results that are shown. It seems that the theoretical concepts of resilience/ resistance are the actual working concepts behind this paper that would make the results better contextualized. The three habitats sampled make a nice gradient for assessing how much things are the same/ different in this system. Rather than focus on issues that you did not measure (i.e. hunting, flooding, etc) keep the introduction more centralized on the things you did measure and it would be a clearer paper.

 In the methods there are number of very large assumptions made of the reader. I previously have read your work on measuring distance to birds from ARUs. While I find the logic of doing this reasonable, I do not remember you having this level of accuracy in distance estimation (i.e. to the metre). It seems like given the novelty of your approach that you need to provide an appendix highlighting how you do this and where this level of accuracy comes from. At a minimum I think you must let the reader know that this is an estimate and recognize there is error because I find it very unlikely that you can do this to a metre for every species.  Thus, some of your birds within the sampling radius were probably past 50 metres and some birds outside 50 were likely excluded. It is likely that such error is random between habitats but you have to make this an explicit assumption.

 The use of 20 minute point counts from the same day is very reasonable for richness and calling rates etc. But I have significant concerns about double counting of the same individual over that duration. Birds move and how a listener can track the number of individuals that occur over that interval is likely very problematic. You need to justify that this did not occur for people to believe this numerical estimates. How you analyzed abundance is unclear regardless (see below).

 In the methods you never use the terms alpha, beta or gamma when describing richness. Yet the figures do. You need to clarify the equations used to calculate beta in particular. How beta is computed needs to be described and the results described given the figure that includes it. No measurement of beta diversity that I have read would have the range you shown in figure 2 so clarification of how is essential.

 I am somewhat confused as to the amount of replication you have. I understand there to be 12 sites. At each site you have 48 hours of data. From that you picked 20 minutes at a set time. You then repeatedly sampled one morning from each month, I think. As such you have repeated measures throughout your design. As it reads, I think your analyses using models with random effects are reasonable. What I am not clear is whether or not the ADONIS test incorporates this repeated measure component. The other issue in analyses that is unclear is you say you use the sum of the maximum number of individuals vocalizing simultaneously. Does this mean you modelled the sum of the max for all repeated surveys? If so then is this a mixed model or are you using the maximum from each count with a random effect for site? Would you not then need to control for month of the year?

 Nowhere do I see rationale for using an 83% confidence interval. It is rather atypical so should be justified.

 The discussion fails to recognize the significant limitation of this study which is replication. You only have 4 replicates for each treatment so the ability to find differences, particularly in alpha is limited. Since most of the other variables build from alpha richness it is not particularly surprising that the null models fit the best. So making a really strong statement that there is no difference is not really a solid argument. There is not a huge effect size clearly, but with 100 replicates in each habitat then I suspect you would support far more complex models and be able to draw stronger conclusions.    

 The discussion also goes into talking about issues of this park that are not presented with the actual results. While these things maybe happening their role in what is described as the question of concern seem like filler rather than important context from the research that was conducted.

I have read this submission. I believe that I have an appropriate level of expertise to confirm that it is of an acceptable scientific standard, however I have significant reservations, as outlined above.

# Response

**Published:** 2018-05-04

**Authors:** Kevin Darras

**Affiliations:** University of Goettingen, Germany

Thank you very much for your critical appraisal of our manuscript. We addressed most of the issues you raised.

 We did not change the focus of the introduction: hunting or poaching is not mentioned in the introduction, and flooding is only mentioned once in the introduction when enumerating the importance of peat swamp forests without further discussion. Wild bird trapping is actually mentioned in the discussion but it is central to it because it poses a direct threat to the bird populations that we surveyed. Flooding is a defining element of swamp forests that the reader needs to bear in mind. That is why we kept both of these themes in the manuscript, even though we did not measure them.

 We do not introduce or develop the resilience concept in this paper as the study was rather designed for documenting the bird communities and to establish their conservation value and as such, it does not address that theme satisfactorily. We think that a much more stringent selection of many more sites would be needed to address this interesting subject. Our sentence “Few studies have focused on the potential benefit of disturbances for overall landscape biodiversity apart from theoretical and modelling approaches ” was changed to “Theoretical and modelling approaches are usually used to analyse the potential benefit of disturbances for overall landscape biodiversity” to avoid implying that we will investigate that topic. We also merged that part with the previous paragraph.

 We clarified in the text that although bird distances were estimated to the metre, accuracy is not as high, as this led to a misunderstanding: “The distance of bird vocalisations was estimated by ear to the meter; even though the accuracy is lower, estimation error was assumed to be random. Distance estimates were made based on the call loudness in the sound recording compared to the ambient sound level and knowledge of the source sound level of each species.“ It is better to do fine-grained estimates even when accuracy is low as estimation errors should be normally distributed and averaged out in the end. Our corresponding manuscript about estimating bird detection distances is in minor revisions.

 We clarified how we counted individuals for our analysis of abundance. We now write “For counting bird abundance, we first derived the maximum number of simultaneously vocalising individuals in each species, and then summed these maxima over all species, leading to a conservative estimate of the number of individuals”. Thus, double-counting individuals was prevented.

 We did not change the statistical models description itself, as it became clearer with the new text that describes how abundance was calculated, and the existing text is straightforward: “At the count level, the species richness and abundance between habitats was modelled using generalised linear mixed effects models of the poisson family (lme4 package^37^), with site as random variable”. We do not use month as an additional random effect as our models would then be over-fitted with one random effect per data point.

 We clarified what our sampling units are and now use the terms “plot” and “site” consistently throughout, as it was sometimes unclear. We also explain better when each plot was sampled: “We recorded audible sound in all 12 plots two times. […] From February to November 2013, we sampled all three plots of one site (one from each habitat) each month for 48 hours, starting at midnight.”

 Concerning alpha and beta richness, we now elaborate how we calculated both richness values in addition to the already cited study about additive richness partitioning: “alpha richness was the mean number of species per plot, and beta richness was defined as the total number of species in the plot’s habitat (gamma richness) minus its alpha diversity.”

 We clarified in the methods that the ADONIS analyses were based on data pooled from both counts, so that we only investigate the general composition of the habitats without considering seasonal effects.

 The rationale behind using 83% confidence intervals is now supported with a proper citation.: Krzywinski, M., Altman, N., 2013. Points of significance: error bars. Nature methods 10, 921–922. This confidence interval value corresponds to intervals that touch each other at a 5% P-value.

 We agree that our spatial replication is limited with only four plots per habitat. However, most ecological studies fail to satisfactorily cover the geographical ranges of the ecosystem of interest, and having more plots was not possible in our case. Moreover, we disagree with the statement that most of our measures build upon alpha richness: abundance, richness, and call activity are only correlated and one cannot replace the other. However our community-weighted means of functional traits are totally independent of richness. Instead, this is precisely the point that we make in our study: even though alpha richness is similar or indistinguishable across sites, we find significant differences in the composition and function of the respective bird communities, underlining the fact that richness (or abundance) does not tell the whole story. We made that clearer in the first paragraph of the discussion that summarises the findings: “This change in the community also became apparent in a shift to bigger and more mobile species with greater distribution ranges in shrub swamp.”

 As you mention, with more replicates we would be able to make finer inferences, and it is indeed our aim to maximise the use of our larger raw data set at hand with future manuscript versions. However, even with our limited data set, the differences we have found so far are statistically significant and thus solid enough to support the message we tell. Still, we added the following sentence to our discussion: “[…] due to logistical restrictions, we only surveyed four sites that were close to waterways, so that that large parts of the park remain little known. “

 We would like to keep the conclusion as is since this study is focused strongly on conservation, and highlighting the threats to this national park and its animals seems to be in line with our message. To develop the wild bird trapping theme further, we added: “Wild bird trapping threatens bird populations directly (Harris, J.B.C., Tingley, M.W., Hua, F., Yong, D.L., Adeney, J.M., Lee, T.M., Marthy, W., Prawiradilaga, D.M., Sekercioglu, C.H., Suyadi, Winarni, N., Wilcove, D.S., 2016. Measuring the impact of the pet trade on indonesian birds. Conservation Biology n/a-n/a. https://doi.org/10.1111/cobi.12729), and is especially worrisome as birds from the Berbak region are increasingly traded in the caged bird market of Jambi city (pers. obs. KD).“

# Peer review: Referee response for version 1

**DOI:** 10.5256/f1000research.15212.r31795

**Published:** 2018-03-12

**Authors:** Badrul Azhar

**Affiliations:** 1 Faculty of Forestry, Institute of Bioscience, Universiti Putra Malaysia, Serdang, Malaysia

Darras et al. present their findings about avian biodiversity in primary swamp forest, secondary swamp forest, and shrub swamp. However, I have some major concerns regarding the way the study was conducted and little information is provide to explain the study areas.

 “We surveyed birds in Berbak National Park, a peat swamp situated in the province of Jambi, on the east coast of the island of Sumatra in Indonesia (Figure 1). Berbak National Park is a Ramsar site25 and an Important Bird Area26. A severe drought in 1997 facilitated forest fires which were aided by human disturbance due to natural rubber collection (Dyera costulata). The burned areas subsequently developed into shrub swamp. Tree stumps also reveal that illegal selective logging affected several areas of the park, resulting in secondary forest habitats.”

 Comments: Provide additional information, size area of each study area? When the secondary forest was logged? Explain about the shrub swamp here.

 “We recorded audible sound in all 12 sites. We used autonomous sound recorders (SM2+ recorders with SMX-II microphones, Wildlife Acoustics Inc.) and did not carry out visual surveys, since acoustic recording constitutes a valid survey method for assessing bird richness27, especially for cryptic birds in tropical forests. From February to November 2013, we sampled all three habitats at one site each month for 48 hours, starting at midnight.”

 Comments: I’m quite confused about the field survey because not much information is provided in the current text. Do you have enough number of spatial replicates to perform GLMMs. And with just data from 12 sites, you can confidently say the results really have answered the research questions. In addition, the sample size is small i.e. 12 sites. Why you did not move the autonomous recorders to other points or sites? You established four sites in each habitat. I think you need to use point method. For example, 30-40 points at each site. I suggest the authors to write a caveat regarding the small sample size in the discussion. The 48 hours were continuous? The sound recording method should be complemented with other sampling methods next time.

 “we counted and measured the diameter at breast height (DBH) of all trees with a circumference above 20 cm (diameter at breast height of ~6.4 cm) inside an area of 14 × 14 m delimited by spanning 10 m coloured ropes from the central tree where the sound recorder was attached to all cardinal directions.”

 Comments: Provide the summary statistics for each variable.

 “For all models, we used tree number, tree basal area, and habitat type as predictors.”

 Comments: Did you include the different sampling month into the modelling work?

 “We detected 379 birds overall, belonging to 90 species.”

 Comments: After doing bird survey for ten months, your results are not very impressive compared to conventional sampling methods. Perhaps you need to have more sites or at least 30-40 points/lines per site. How many endangered/vulnerable species were recorded?

 References

 Comments: The reference list is not comprehensive. Authors should search and cite recent published bird studies in tropical peat swamp forest of SEA. Some articles cited in the text are not about studies from tropical peat swamp forests.

I have read this submission. I believe that I have an appropriate level of expertise to confirm that it is of an acceptable scientific standard, however I have significant reservations, as outlined above.

# Response

**Published:** 2018-05-04

**Authors:** Kevin Darras

**Affiliations:** University of Goettingen, Germany

Thank you very much for your critical appraisal of our manuscript. We addressed most of the issues you raised.

 We added the total size of the national park to the legend of figure 1 (1476 km2). Each one of our plots was a point, and we used the birds within a 50 m radius of that point for our analysis, as explained in the methods. We stated ****until when the secondary forest sites were logged and the shrub bush burned: “The secondary forest plots were last subject to selective logging until 2008 for BS1, 1999 for BS2, 2003 for BS3, and 2010 for BS4. The shrub swamp sites burned in 1997, and BB2 burned yearly since.”.

 We provided summary statistics for the total DBH in each habitat in the Fig S2 caption: 89 m2 per ha in primary forest, 64 m2 per ha in secondary forest, and 29 m2 per ha in shrub swamp.

 We also clarified the data collection schedule, as this also explains why we could not systematically test for the seasonal effect in our models: “From February to November 2013, we sampled all three plots of one site each month for 48 hours, starting at midnight. It was not possible to do the survey each month, or to repeat the same site sampling sequence for the second set of recordings because of access and transportation restrictions.”

 Also, we did not use the sampling month as a predictor in our models as we only sampled 4 sites each month and that effect would be confounded with the spatial effect from the 4 plots being close to each other.

 We did not move the recorders to other sites because it would have been difficult to do so. The park is very difficult to reach and go through, and we could not do more than what we achieved in 8 months of sampling. Also, we lacked skilled ornithologists to carry out point counts and it would have been prohibitively expensive. To get an impression of the field work, you can have a look at our blog post here: https://blog.f1000.com/2018/04/10/can-hear-birds/

 We now cite the study of Azhar et al. 2011 in the introduction and also in the discussion about the secondary forest value: “It is noteworthy that even extensively logged peat swamp forest can harbor large species numbers.(Azhar, B., Lindenmayer, D.B., Wood, J., Fischer, J., Manning, A., McElhinny, C., Zakaria, M., 2011. The conservation value of oil palm plantation estates, smallholdings and logged peat swamp forest for birds. Forest Ecology and Management 262, 2306–2315.).”

 We would like to point out that the statistical analysis has been explained: “At the count level, the species richness and abundance between habitats was modelled using generalised linear mixed effects models of the poisson family (lme4 package ^37^ ), with site as random variable [...]”. Since we have two counts (=data points) per site, mixed models were adequate. Also, the number of replicates was sufficient for proving our points about the functional changes observed in the bird communities of the different habitats, since our statistical analysis yielded significant P-values, even when using these stringent mixed effects models.

 The 48 hours were continuous indeed, and we will process more data as soon as we find people interested in doing this. 387 birds found in 8 months is not impressive *per se* indeed, but please bear in mind that this is the result of analysing only 0.7% of our data. We also updated these numbers by better counting of simultaneous detections, yielding a data set of 426 birds (of which 394 were used in the analysis after discarding far and unidentified birds).

 We added the following sentence to our discussion: “[…] due to logistical restrictions, we only surveyed four sites that were close to waterways, so that that large parts of the park remain little known. “
